# Non-Coding RNAs in the Regulation of Doxorubicin-Induced Cardiotoxicity

**DOI:** 10.3390/biom15060800

**Published:** 2025-05-31

**Authors:** Mengyao Sun, Il-Man Kim, Lei Yang

**Affiliations:** 1 Department of Pediatrics, Indiana University School of Medicine, Indianapolis, IN 46202, USA; 2 Department of Anatomy, Cell Biology, and Physiology, Indiana University School of Medicine, Indianapolis, IN 46202, USA

**Keywords:** doxorubicin, cardiotoxicity, non-coding RNAs, microRNAs, long non-coding RNAs, circular RNAs, cell death

## Abstract

Doxorubicin, a commonly prescribed chemotherapeutic drug in clinical practice, is associated with severe cardiotoxicity that restricts its long-term use in cancer treatment. Recent studies have highlighted the critical roles of non-coding RNAs (ncRNAs) in the regulation of doxorubicin-induced cardiotoxicity (DIC). Notably, ncRNAs, including microRNAs, long non-coding RNAs, and circular RNAs, display critical functions in various DIC-associated cellular processes, such as cell death, oxidative stress, and mitochondrial dysfunction, all of which contribute to the pathophysiology of DIC. Accumulated evidence indicates that ncRNAs regulate gene expression by interacting with DNAs, RNAs, proteins, and lipids, presenting a potential avenue to alleviate the adverse effects of doxorubicin on hearts. This review discusses the emerging research progress focusing on the molecular mechanisms by which ncRNAs regulate DIC. Understanding the complicated and essential roles of ncRNAs in DIC could thus pave the way for developing novel cardioprotective strategies.

## 1. Introduction

Doxorubicin (DOX), a member of the anthracycline family of chemotherapeutic agents, has been widely used in clinics for treating various malignancies, including breast cancer, lymphomas, and leukemia [1]. Despite the prominent anti-tumor effect of DOX, doxorubicin-induced cardiotoxicity (DIC) prevents the long-term administration of DOX for cancer therapy [2]. Remarkably, DIC eventually leads to irreversible cardiac dilated cardiomyopathy and congestive heart failure (CHF) [2]. The underlying pathogenesis of DIC involves complex mechanisms that contribute to cardiac injury [3], including DNA damage, excessive generation of reactive oxygen species (ROS), mitochondrial dysfunction, impaired calcium homeostasis and cell death [4,5,6,7,8]. Currently, given the serious risk of DIC in cancer therapy and the complicated interactions between tumor and heart upon DOX treatment, there is an urgent need to deeply uncover novel molecular mechanisms underlying DIC and develop effective strategies to mitigate DIC while maintaining the anticancer efficacy of DOX.

Over the past decades, research on DIC has primarily focused on the identification of coding gene-related mechanisms and pathways in cardiac injury, such as mitochondrial dysfunction [6], excessive ROS [5], Topoisomerase II beta (TOP2B) inhibition [4] and cell death including apoptosis, ferroptosis, pyroptosis, and autophagy [9] in the development of DIC. Notably, recent studies have uncovered the significant impacts of non-coding RNAs (ncRNAs) on the pathophysiology of DIC and revealed the promising potential of ncRNAs as therapeutic targets of DIC [10]. Studies of ncRNAs, including microRNAs (miRNAs), long non-coding RNAs (lncRNAs), and circular RNAs (circRNAs), represent a new frontier in understanding the molecular etiology of DIC and offer novel targets for potential diagnostic and therapeutic interventions. This review summarizes the current mechanistic findings of three types of regulatory ncRNAs, including miRNAs, lncRNAs, and circRNAs, in DIC.

## 2. Mechanisms of Dox-Induced Cardiotoxicity

### 2.1. DNA Damage-Induced Apoptosis

The p53 is a key regulator of cell death and apoptosis, which plays an important role in DOX-induced cardiomyocyte loss. It has been reported that DOX can upregulate p53 in response to enhanced oxidative stress, which increases proapoptotic gene expression and promotes cardiomyocyte death [11]. Zhang et al. reported that TOP2B is the major target of DOX in cardiomyocytes [4]. DOX covalently binds to and inhibits the function of TOP2B, which is an enzyme crucial for DNA replication, transcription, and repair, leading to DNA double-strand breaks within cardiomyocytes. DOX interacts with TOP2B to trigger DNA damage, which activates the tumor suppressor protein p53 [4]. Once activated, p53 triggers a cascade of events that leads to the initiation of apoptosis in cardiomyocytes, ultimately leading to cardiac muscle loss [4].

### 2.2. Mitochondrial Dysfunction and ROS Production-Related Apoptosis

Mitochondria play a key role in cardiomyocytes, which produce ATP for cardiomyocyte contraction and maintain other cellular functions. Increasing evidence indicates that DOX can directly interfere with mitochondria in cardiomyocytes, causing a detrimental effect on mitochondria [12,13]. Given its cationic property, DOX can integrate into the mitochondrial membrane and form an irreversible complex with the anionic phospholipid called cardiolipin, an essential component of the mitochondrial cristae membranes [14]. Cardiolipin is critical for maintaining the proper function of the electron transport chain (ETC) to produce ATP [14]. The tight interaction of DOX with cardiolipin can disrupt the function of cardiolipin at the interface. Additionally, the metabolites of DOX accumulated in the inner mitochondrial membrane could be utilized by the complex I of the ETC, leading to the production of excessive ROS and consequent oxidative damage to the ETC complexes [14,15,16]. The binding of DOX to cardiolipin can also reduce the pool of cardiolipin that is essential for anchoring cytochrome c in mitochondria, which causes the release of cytochrome c from mitochondria into the cytoplasm to trigger apoptosis [17].

Moreover, DOX represses long-chain fatty acid oxidation (FAO) in mitochondria while increasing glucose metabolism of cardiomyocytes, promoting anaerobic metabolism alongside reducing aerobic metabolism [3]. This metabolic shift contributes to the impairment of cardiac contractility and relaxation and further exacerbates heart failure [12,18]. Additionally, DOX can reduce the expression level of the transcription factor GATA binding protein 4 (GATA4) in cardiomyocytes [3]. GATA4 is the upstream factor that activates the antiapoptotic gene B-cell lymphoma extra large (Bcl-XL) [19,20]. Therefore, depletion of GATA4 by DOX can lead to the augmentation of mitochondrial damage-induced apoptosis in cardiomyocytes [3,19].

### 2.3. Ferroptosis

In addition to apoptosis, DNA damage and mitochondrial dysfunction can induce other forms of regulated cell death (RCD), such as ferroptosis and pyroptosis, which have been identified in DIC [21]. Ferroptosis is an iron-dependent, nonapoptotic cell death, which is characterized by cell swelling, condensed mitochondria and distorted mitochondrial cristae. Since ferroptosis was identified in 2012 [22], increasing studies have reported its critical role in the pathological progression of DIC.

Ferroptosis is characterized by iron-dependent lipid peroxidation, which is mainly regulated by iron, lipid, and glutathione (GSH)/glutathione peroxidase 4 (GPX4) metabolism [21]. Under normal conditions, two ferric irons (Fe^3+^) carried by one transferrin (TF) are transported into cells through transferrin receptor protein 1 (TFRC), and the intracellular iron is primarily stored in ferritin, which protects cells from damage induced by excessive iron [23]. DOX can enhance the expression level of TFRC, leading to iron overload into cells [21]. Additionally, DOX has a strong affinity for iron, forming a DOX–iron complex in cardiomyocytes [24]. Additionally, DOX treatment results in nuclear factor erythroid 2-related factor 2 (Nrf2)-dependent heme oxygenase 1 (Hmox1) gene upregulation. The enzyme Hmox1, coded by Hmox1 gene, can mediate heme degradation, thereby increasing the release of iron into the cytosol [25,26,27]. These mechanisms contribute to the increased labile iron pool (LIP) in cardiomyocytes, thereby inducing ferroptosis through the Fenton reaction [21]. Furthermore, in the lipid peroxidation process, DOX activates acyl–coenzyme A synthase long-chain family member 4 (ACSL4), contributing to lipid peroxidation, and lipid peroxides can produce cytotoxic 4-hydroxynonenal (4-HNE) and malondialdehyde (MDA) [23]. Finally, the cystine/glutamate antiporter (xCT)/GSH/GPX4 pathway suppresses ferroptosis [21]. The xCT takes in cystine for GSH biosynthesis [21]. GPX4 suppresses lipid peroxidation in the presence of GSH. GPX4 is downregulated upon DOX treatment and thus results in increased lipid peroxidation and ferroptosis [27].

### 2.4. Pyroptosis

Pyroptosis is a type of programmed cell death identified in 2001 by Drs. Brennan and Cookson [28]. It is now commonly considered a crucial type of RCD in the pathogenesis of cardiovascular diseases. Although pyroptosis has been investigated in various diseases, it was not demonstrated in DIC until 2019 by Meng et al. [29]. Pyroptosis is a type of inflammatory programmed cell death and is characterized by cell swelling, membrane rupture, and release of pro-inflammatory molecules. The activation of caspase-1, caspase-4, caspase-5, and caspase-11 as well as NLR family pyrin domain containing 3 (NLRP3) in pyroptosis leads to the cleavage of gasdermin D (GSDMD) or GSDME and the formation of transmembrane pores that allow the release of interleukin-1beta (IL-1β) and IL-18 [30].

In DIC, DOX triggers pyroptosis through the upregulation of Terminal Differentiation-Induced Non-Coding RNA (TINCR), and TINCR recruits insulin-like growth factor 2 mRNA-binding protein 1 (IGF2BP1) to enhance NLRP3 transcription [29]. The effect of TINCR on cardiomyocyte pyroptosis could be attenuated by the inhibition of NLRP3 or IGF2BP1 [29]. It was reported that increased levels of cleaved caspase-3, caspase-1, IL-1β, IL-18 and GSDMD-N were associated with pyroptosis in DIC [29], and GSDMD could mediate pyroptosis in DIC by directly binding with DOX [31]. Additionally, GSDMD regulates DOX-induced mitochondrial damage via Bcl-2 interacting protein 3 (Bnip3) and mitochondrial perforation in cardiomyocytes [31]. Furthermore, Ye et al. reported that emodin could directly bind GSDMD to attenuate pyroptosis in DIC [32].

### 2.5. Autophagy

In eukaryotic cells, autophagy is a conserved process that maintains cellular homeostasis and survival under normal and stressful conditions. It removes aged and damaged organelles and proteins under both physiological and pathological stimulations. The autophagy process in the heart can be dysregulated upon stress and thus cause cardiac dysfunction and heart failure [33,34]. DOX can alter the expression levels of key genes related to autophagy, thereby resulting in cardiotoxicity in DIC. While some researchers observed reduced autophagy in DIC, others found the opposite. For instance, Liang et al. demonstrated that DOX treatment induced autophagy probably through the depletion of transcription factor GATA4, which reduced the expression of antiapoptotic protein B-cell leukemia/lymphoma 2 (Bcl-2), leading to cell death [35]. Additionally, another study reported that DOX could activate autophagy by increasing the phosphorylation of Bcl-2, which inhibits the interaction of Bcl-2 with Beclin1 [35,36]. Bcl-2 can bind and suppress Beclin1 activity to inhibit its role in autophagy [37]. Furthermore, LC3B, a marker of autophagy, is upregulated by DOX administration to induce cardiac damage [38]. On the other hand, Jordan et al. reported that DOX administration triggered the accumulation of autophagosomes and inhibition of autophagic flux through downregulation of the transcription factor EB (TFEB) [39]. TFEB is a master regulator of autophagy and lysosomal function, and its loss is responsible for the reduction of several autophagy-related gene (Atg) expression and lysosomal proteolytic activity, contributing to cell death [40]. Moreover, the accumulation of autophagosomes results in ROS generation [41,42,43]. DOX administration upregulates the expression of genes including Atg5, Atg12, Atg4 and Bad and downregulates Beclin1. In contrast, curcumin protects cardiomyocytes from DOX-induced damage by blocking apoptosis and enhances autophagy by regulating the c-Jun N-terminal kinases (JNK)-mediated pathway [38,44]. Altogether, DOX-impaired autophagy can play a vital role in cardiomyocyte death.

## 3. Non-Coding RNAs involved in the DIC

### 3.1. MicroRNAs and DOX-Induced Cardiotoxicity

#### 3.1.1. Introduction to MicroRNAs

**Figure 1:**
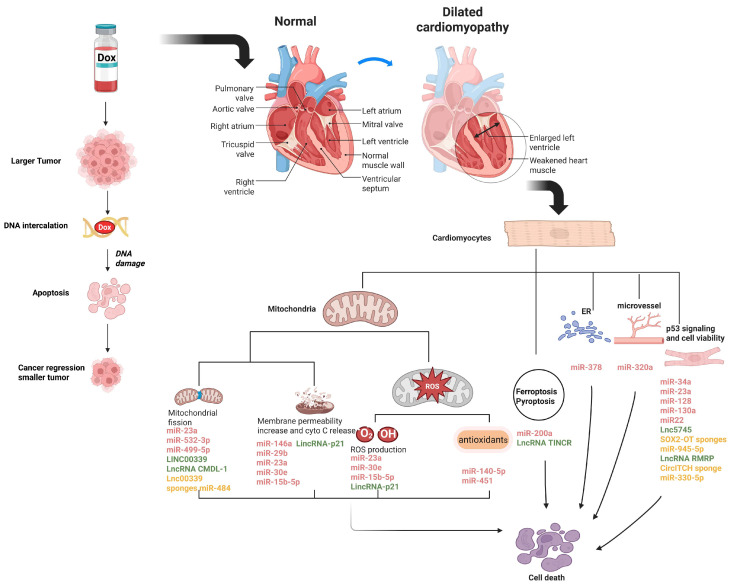
NcRNAs involved in the regulation of DIC. Schematic diagram represents the molecular mechanisms of most of the discussed ncRNAs in regulating DIC. MiR-499-5p, miR-23a, miR-532-3p, lnc00339 and lnc CMDL-1 are involved in DOX-induced mitochondrial fission; MiR-146a, miR-30e, miR-29b, miR-23a, miR-15b-5p and lincRNA-p21 participate in the reduction of mitochondrial membrane potential and release of cytochrome c induced by DOX; MiR-30e, miR-23a, miR-15b-5p and lincRNA-p21 regulate DOX-induced ROS production; MiR-140-5p and miR-451 are related to DOX-altered antioxidant levels; MiR-378 is associated with DOX-induced ER stress; MiR-320a regulates microvessel density under DOX treatment; MiR-34a, miR-23a, miR-128, miR-130a, miR-22, miR-23a, lnc5745, and lncRNA RMRP are correlated with the p53 signaling pathway; and MiR-200a is related in DOX-induced ferroptosis, while lncRNA TINCR has an important role in pyroptosis of DIC. Some lncRNAs and circRNAs can sponge miRNAs. The figure was created in Biorender (https://BioRender.com) by M.S. (2025).

MicroRNAs (miRNAs) are a class of small endogenous single-stranded ncRNAs that are ~20 nucleotides in length. Some miRNAs are conserved across species [45]. MiRNAs bind to their target messenger RNAs (mRNAs) through base pairing, which affect mRNA stability and/or translation [46,47]. Clinical trials and animal experiments indicate that miRNAs are involved in various cardiovascular diseases, such as coronary heart disease, ischemia-reperfusion injury, heart failure and DIC [48,49,50,51]. Altered expressions of miRNAs have been reported in DIC [48,52,53] (Figure 1), which subsequently cause the aberrant expressions of target genes in cardiomyocytes, inducing apoptosis, mitochondrial dysfunction, ROS and/or autophagy (see Table 1).

#### 3.1.2. MiRNAs Directly Modulate p53 Signaling-Related Genes to Affect Cardiomyocyte Apoptosis in DIC

Since cardiomyocytes have limited regeneration capacity after DOX treatment, previous studies suggested that apoptosis was the predominant cellular event in DIC [100,101]. Among the molecular pathways and mechanisms underlying apoptosis in DIC, p53- and mitochondria-dependent apoptotic pathways have been extensively studied.

It was reported that miRNAs can directly interfere with p53 signaling-related genes to regulate apoptosis of cardiomyocytes after DOX treatment [102]. For example, Piegari et al. and Zhu et al. observed upregulated expression of miR-34a in rat cardiomyocytes after DOX treatment and reported that pro-survival gene Sirtuin 1 (SIRT1) was a direct target of miR-34a [61,62]. De Angelis et al. [103] reported that DOX could induce acetylation of p53 in human cardiac progenitor cells, promoting cell cycle arrest and apoptosis.

Piegari et al. validated the positive feedback loop of p53-miR-34a-SIRT1 axis in a rat model: DOX stressed cardiac cells and activated p53, p53 induced miR-34a, and miR-34a maintained p53 acetylation by inhibiting the expression of SIRT1 [61]. They also found that antimiR-34a treatment suppressed apoptosis of cardiomyocytes via upregulating antiapoptotic Bcl-2 and SIRT1 [61]. Similarly, Zhu et al. found that miR-34a level was increased in rat cardiomyocytes after DOX exposure, which was reversed by dexrazoxane [62]. They proposed that the upregulated miR-34a in DIC resulted from the activation of Nuclear Factor kappa B (NF-κB) and p53 signaling pathways and that miR-34a inhibited SIRT1, leading to apoptosis by enhancing expression of the proapoptotic gene p66shc (SHC adaptor protein 1) [62]. Therefore, both studies demonstrate that miR-34a plays a proapoptotic function in DIC by directly binding to the mRNA of pro-survival gene SIRT1.

Additionally, miR-23a and miR-128 were associated with p53 regulation in DIC [57,63]. MiR-23a was upregulated by hydrogen peroxide and DOX, which sensitized cells toward apoptosis by binding to p53 [57]. An increased miR-128 level was found in DOX-treated hearts and cardiomyocytes [64]. MiR-128 promotes apoptosis by directly binding with SIRT1, suppressing its expression and thereby increasing p53 acetylation and activity [63,64]. Additionally, miR-128 could directly target peroxisome proliferator-activated receptor γ (PPAR-γ), a cardioprotective gene against oxidative stress and apoptosis, to repress DIC [64,104]. Another microRNA that could target PPAR-γ in DIC is miR-130a [65]. Pakravan et al. showed a sharp increase of miR-130a expression after DOX treatment, and bioinformatics analysis predicted that PPAR-γ could be a direct target of miR-130a in DIC [65], although the prediction was not functionally validated. MiR-22 was found to play a role in DIC via the p53 signaling pathway [56]. DOX induced miR-22 upregulation in murine cardiomyocytes, while miR-22 inhibition mitigated DIC via directly targeting the 3′UTR of SIRT1 [56].

#### 3.1.3. MiRNAs Directly Regulate Mitochondria-Dependent Apoptotic Genes in DIC

Since mitochondria are enriched in cardiomyocytes [5,6], mitochondria-dependent apoptosis has been widely investigated in DIC. DIC is closely associated with mitochondrial morphological change and dysfunction. DOX binds to mitochondrial cardiolipin and forms a DOX–cardiolipin complex. This complex disrupts the ETC and reduces the available cardiolipin for stabilizing cytochrome c within mitochondria [105]. Therefore, DOX induces the release of cytochrome c from mitochondria into the cytoplasm, which subsequently activates apoptotic protease activating factor-1 (Apaf-1), ATP/dATP and caspase-9, triggering the caspase cascade that ultimately leads to apoptosis of cardiomyocyte [106]. Additionally, DOX may directly intercalate into mitochondrial genome—mtDNA—to form adducts [107], which disturb the functions of mitochondrial proteins and impair mitochondrial functions [107].

Mitochondrial fission and fusion are critical for maintaining the normal function of mitochondria [108]. Excessive fission can contribute to cardiac injury under stress conditions, such as ischemia and DOX treatment [109,110]. A previous study identified the role of miRNA-532-3p in regulating mitochondrial fission in DIC [48]. Upon DOX treatment, miR-532-3p is upregulated to promote DOX-induced mitochondrial fission and apoptosis by directly targeting apoptosis repressor with caspase recruitment domain (ARC), an apoptosis suppressor [48]. It was also reported that ARC was downregulated in pathological heart conditions, whereas the overexpression of ARC inhibited DOX-induced mitochondrial division, thereby attenuating myocardial apoptosis [48]. Another study showed that miR-499-5p regulated mitochondrial fission during myocardial infarction [73]. Interestingly, miR-499-5p expression was reduced upon DOX treatment, which led to the upregulation of its target p21, a transcription factor involved in heart injury, which subsequently induced mitochondrial fission and apoptosis in DIC [74]. In addition, enhanced miR-499-5p expression promoted cardiac function, suggesting that the downregulated miR-499-5p may contribute to cardiac dysfunction in DIC [74].

Functionally, the loss of mitochondrial membrane permeability and cytochrome c release are the two main characteristics of DOX-induced mitochondrial injury [6,107]. Heart-abundant miR-146a, which was upregulated by DOX in rat neonatal cardiomyocytes, was responsible for the reduced mitochondrial membrane potential and myocardial apoptosis in acute DIC by reducing Erb-B2 Receptor Tyrosine Kinase 4 (ErbB4), while inhibition of miR-146a and overexpression of ErbB4 could attenuate DIC [66]. However, this study did not detect miR-146a expression in vivo nor explore the roles of DOX-triggered miR-146a upregulation and ErbB4 downregulation in vivo [66]. In 2019, Gu et al. explored the role of miR-146a in a chronic DOX-induced cardiotoxicity model and reported that overexpression of miR-146a can reverse apoptosis and rescue autophagy through the TATA-binding protein (TBP)-associated factor 9b (TAF9b)/p53 pathway [67]. TAF9b is a coactivator and stabilizer of p53, which stabilizes the structure of proapoptotic p53. The upregulated miR-146a targeted TAF9b to inhibit its activity, thereby downregulating p53, reducing apoptosis and improving autophagy in cardiomyocytes [67]. Notably, this research group utilized a chronic DIC model, so they observed that the expression of miR-146a first rose then descended. The reason why these two research groups reached different conclusions about miR-146a may be because the former group focused on the acute or early stage of DIC, whereas the other group paid more attention to the late stage of DIC. Moreover, another study found that DOX suppressed miR-29b expression in cardiomyocytes, which negatively affected the expression of proapoptotic protein Bax by directly binding its 3′UTR region, activating the mitochondria-mediated apoptotic pathway [59]. MiR-29b agomir ameliorated DOX-induced cardiac injury. Although the differential miR-146a expression in DIC has been observed using different animal models, these studies imply an important implication of miR-29b for treating DIC [59].

#### 3.1.4. MiRNAs and DOX-Induced Oxidative Stress in Apoptosis

Mitochondria and oxidative stress interconnect to create a feedback loop of detrimental effects in DIC. Metabolism of DOX creates unstable DOX-semiquinone radicals, which are then re-oxidized back to DOX and ROS can be generated in this procedure [5]. In the redox cycle, ROS are continuously produced, which interact with intracellular components to induce oxidative damage to biological macromolecules and organelles, eventually resulting in tissue injury [111,112]. Mitochondria are the major organelles in the heart where ROS are produced, and the binding of DOX to mitochondrial cardiolipin inhibits the activity of complex I and disrupts the ETC, resulting in increased ROS production [5]. Mitochondrial DNA (mtDNA) damage could be induced by elevated ROS, leading to the downregulation of ETC proteins encoded by mtDNA and exacerbated mitochondrial dysfunction [113]. In addition, excessive ROS can alter the mitochondrial respiration rate and, together with Ca^2+^, enlarge the opening of the mitochondrial permeability transition pore (mPTP), resulting in the loss of mitochondrial membrane potential and the release of cytochrome c to activate the apoptotic signaling cascades [114].

The involvement of miR-15b-5p and miR-23a in DOX-induced apoptosis of cardiomyocytes was attributed to the mitochondria-related pathways as well as ROS-related mechanisms. The upregulation of miR-15b-5p significantly enhanced the detrimental effects of DOX on the permeability of the mitochondrial membrane and ROS production [54]. Both miR-15b-5p overexpression and the inhibition of its target, Bmpr1a, exacerbated DOX-induced apoptosis of cardiomyocytes by reducing the ratio of Bcl-2/Bax (an antiapoptotic indicator) [54]. Similarly, DOX treatment upregulated miR-23a, which induced excessive mitochondrial fission, leading to enhanced phosphorylation of dynamin-related protein-1 (Drp1), suppressed mitofusin 2 (MFN2) expression, and mitochondria-dependent apoptosis [58]. A miR-23a inhibitor elicited protective effects on DOX-treated cardiomyocytes by restoring the mitochondrial membrane potential and suppressing oxidative stress by targeting peroxisome proliferator-activated receptor gamma coactivator-1α (PGC-1α) and repressing Drp1 phosphorylation [58]. However, the impacts of miR-15b-5p and miR-23a on cardiac function have not been fully investigated. DOX treatment significantly downregulated the expression of miR-30a, miR-30d, and miR-30e in cardiomyocytes, which share the same core seed sequences and could target the same genes. Of the three miR-30s, miR-30e shows the most dramatic downregulation by DOX and can target genes including β1AR, β2AR, Giα-2 and Bcl-2 interacting protein 3 like (Bnip3L) [60]. Activated Bnip3 expression is responsible for mPTP opening, the loss of mitochondrial membrane potential and ultimately cardiomyocyte death [115]. Hence, overexpression of miR-30 reduces the DOX-induced ROS production and the ratio of Bax/Bcl-2 protein, and caspase activity, indicating the beneficial effect of increased miR-30 levels in mitigating DIC [60].

Moreover, DOX also remarkably decreases endogenous antioxidants, which attenuate oxidative stress. Therefore, the administration of antioxidants shows a beneficial effect in DIC [116,117]. In vitro and in vivo studies revealed that DOX elevated the expression level of miR-140-5p and reduced the expression of its target genes, including Nrf2 and sirtuin 2 (SIRT2), both regulating oxidative stress through binding to antioxidant response elements or activating forkhead box class O 3a (FOXO3a) [52]. Administration of a miR-140-5p agomiR to DOX-treated mice led to reduced superoxide dismutase (SOD) activity, whereas the antagomiR had no effect on SOD activity or cardiac function. This suggests that miR-140 upregulation may play a key role in modulating oxidative stress in DIC [52]. More recently, another study demonstrated that inhibiting miR-451 restored antioxidant SOD activity and protected cardiomyocytes from DOX-induced apoptosis by activating the AMPK signaling pathway. Furthermore, miR-451 inhibition alleviated DOX-induced cardiac dysfunction [72].

#### 3.1.5. MiRNAs and Ferroptosis

Ferroptosis plays a key role in DIC, and the inhibition of ferroptosis and lipid peroxidation could effectively protect cardiomyocytes in DIC. Two groups reported that miR-200a could display a cardioprotective role in ferroptosis triggered by DOX via Nrf2 [68,69]. Wang et al. also found that taxifolin can relieve DOX-induced ferroptosis in mouse hearts through the miR-200a-Nrf2 axis [68].

#### 3.1.6. MiRNAs and Endoplasmic Reticulum (ER) Stress-Related Apoptosis Induced by DOX

The ER is a cellular organelle responsible for protein folding and calcium regulation. Under DOX treatment, ER stress arises from the disruption of protein folding and calcium homeostasis. The accumulation of misfolded proteins may generate ROS and altered calcium levels may result in mPTP opening and mitochondrial potential loss [118,119]. Wang et al. reported that miR-378 exerted a cardioprotective effect by modulating energy metabolism and ER stress. It alters mitochondrial membrane potential, which in turn downregulates the expression of mitochondria-associated proteins, such as lactate dehydrogenase A (LDHA), and ER stress-related genes, including cyclophilin A (PPIA) [53]. Consequently, miR-378 enhances cardiomyocyte viability and inhibits apoptosis [53].

#### 3.1.7. MiRNAs and Other DOX-Induced Mechanisms

Beyond the above-mentioned mechanisms, the pro-hypertrophic miR-212/132 family attenuated DOX-induced apoptosis and atrophy in primary rodent- and human-induced pluripotent stem cell (hiPSC)-derived cardiomyocytes [120]. In vivo overexpression of the miR-212/132 cluster improved left ventricular ejection fraction after DOX treatment. In addition, the wall thickness of the ventricle was dramatically decreased by DOX, a parameter for atrophy, which was alleviated by enhancing the miR-212/132 expression level. These findings revealed that downregulated miR-212/132 levels may be related to DIC. At the cellular level, overexpression of the miR-212/132 cluster reduced the rate of apoptosis by directly enhancing its downstream target fat storage-inducing transmembrane protein 2 (Fitm2), which localizes in the ER and is involved in lipid droplet accumulation [120]. Although miR-21, miR-34a-5p, miR-130a, and miR-208a were also involved in the regulation of DIC, no detailed mechanisms were investigated (Table 1) [55,62,65,70]. Additionally, DOX-induced downregulation of let-7g may contribute to the development of DIC [75].

Despite the focus on cardiomyocytes in DIC, Yin et al. investigated DIC in vascular endothelial cells and its impact on vascular homeostasis. MiR-320a was firstly reported to contribute to atherogenesis, which was significantly increased in human umbilical vein endothelial cells (HUVECs) compared to H9c2 cells upon DOX treatment [71]. DOX augmented the rate of apoptosis, suppressed proliferation of HUVECs, and impaired endothelial cell function. Inhibition of miR-320a was found to attenuate DOX-induced endothelial cell injury and increase microvessel density by targeting vascular endothelial growth factor (VEGF)-A, a key regulator of vascular homeostasis, especially in regulating neovascularization [71]. Endothelial cells support cardiomyocyte survival through the paracrine secretion of vascular bioactive molecules [121]. For example, DOX significantly reduced endothelial cell-derived nitric oxide (NO) levels in both HUVECs and mouse hearts, whereas restoration of cardiac NO levels preserved cardiac function in DOX-treated mice [71,122]. Besides that, endothelial cells serve as a barrier, protecting cardiomyocytes from the exposure to harmful substances. However, DOX disrupts the tight junctions to impair the integrity of the endothelium, resulting in an increased microvascular permeability that significantly impairs cardiac contractility and function [123,124]. Therefore, microvascular injury may precede cardiac dysfunction in DIC and contribute to the development of DIC. In addition, suppression of miR-320a improved the cardiac function of DOX-treated mice, suggesting that miR-320a may significantly contribute to the severity of DIC [71].

### 3.2. Long Non-Coding RNAs (LncRNAs) and DOX-Induced Cardiotoxicity

#### 3.2.1. Introduction of lncRNAs

**Figure 2:**
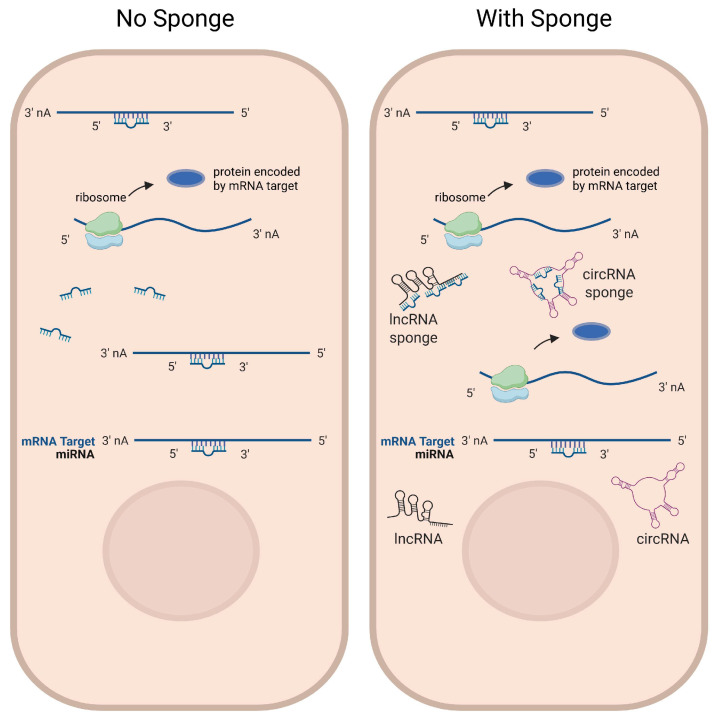
LncRNAs and circRNAs function as miRNA sponges. Some lncRNAs and circRNAs contain multiple binding sites for miRNAs to function as miRNA sponges, which compete with miRNA-targeted mRNAs for miRNA binding. **Left**: in the absence of sponge RNA expression, target mRNAs are post-transcriptionally repressed by the miRNA. **Right**: in the presence of sponge RNA expression, target mRNAs are less repressed by miRNAs, potentially leading to increased expression levels of target genes. Figure is created in Biorender (https://BioRender.com) by M.S. (2025).

LncRNAs are composed of over 200 nucleotides with limited coding potential [125]. LncRNAs exert their biological functions mainly at the epigenetic, transcriptional, and post-transcriptional levels. LncRNAs can modulate the localization and function of epigenetic and transcriptional factors [126]. Additionally, lncRNAs can act as “miRNA sponges/decoys”, binding to miRNAs as competitive endogenous RNAs (ceRNAs) (Figure 2). This reduces the availability of miRNAs to target downstream mRNAs, potentially influencing the expression of target genes in various physiological processes [127,128]. Recently, accumulated evidence indicated that lncRNAs play critical roles in cardiac development and disease [129,130]. We summarize the currently known functions of lncRNAs in regulating DIC in Table 1.

#### 3.2.2. LncRNAs Regulate DIC

Similar to miRNAs, lncRNAs regulate DIC through affecting mitochondrial function and ROS generation. LncRNA NORAD regulates DIC via affecting mitochondrial ROS production, fission, and autophagy (p53-Parkin) pathways both in vivo and in vitro [77]. Long intergenic non-coding RNA (LincRNA)-p21 plays a regulatory role in DOX-induced cardiomyopathy, and suppression of lincRNA-p21 attenuates the DOX-induced loss of mitochondrial membrane potential, oxidative stress and cardiomyocyte senescence by negatively modulating the β-catenin pathway [87].

Regulation of cardiomyocyte death remains the key focus of lncRNAs in DIC. Overexpression of lncRNA NONMMUT015745 (lnc5745) could alleviate DOX-induced apoptosis of cardiomyocytes both in vitro and in vivo [76]. Lnc5745 protects cardiomyocytes against DOX-induced apoptosis through suppressing Rab2A expression and modifying p53 phosphorylation, thereby regulating the p53-related apoptotic signaling pathway [76]. Upon DOX treatment, LncRNA LINC00339 was upregulated, which enhanced DOX-induced apoptosis by competitively sponging heart-enriched miR-484 that inhibited mitochondrial fission and apoptosis [78]. The LncRNA cardiac hypertrophy-related factor (CHRF), well-known for its role in regulating cardiac hypertrophy, was found to be upregulated following DOX treatment. Inhibition of CHRF alleviated DOX-induced cardiomyocyte apoptosis through modulating the TGF-β/Smads and TGF-β/p38 signaling pathways [88]. Interestingly, the expression of lncRNA Mhrt was significantly reduced in cardiomyocytes after DOX exposure. This was also observed under other cardiac stress conditions. Overexpression of lncRNA Mhrt promoted the recruitment of H3 histone to the Nrf2 promoter, enhancing Nrf2 expression and thereby mitigating DOX-induced apoptosis [90]. Furthermore, both lncRNA FOXC2-AS1 and WNT1-inducible signaling pathway protein-1 (WISP1) were downregulated in DIC [89]. Overexpression of FOXC2-AS1 improved cardiomyocyte viability by increasing WISP1 upon DOX treatment. However, the intermediators involved in the FOXC2-AS1- and WISP1-mediated regulation of DIC remained largely unexplored [89]. Aung et al. reported that mitochondrial lncRNA CMDL-1 may play an anti-apoptotic role in DIC by regulating Drp1 phosphorylation at S637 [81]. Wang et al. observed that knockdown of SOX2-OT downregulated DP5 via sponging miR-942-5p and inhibiting DOX-induced apoptosis in primary cardiomyocytes [83]. Gong et al. showed that overexpression of lncRNA RMRP could inhibit the expression of p53 and its phosphorylation level by suppressing profilin 1 (PFN1) to exert cardioprotective effects, holding great promise for serving as a therapeutic target and potential biomarker of DIC [86]. In the DIC in vitro model, DOX treatment resulted in the upregulation of methyltransferase-like 14 (METTL14), which catalyzed the m6A modification of lncRNA KCNQ1OT1, a miR-7-5p sponge [79]. MiR-7-5p has been discovered to bind to the 3′-UTR of the TFRC and controls its expression [79]. A lack of miR-7-5p expression led to increased levels of TFRC, promoting the uptake of iron and production of lipid reactive oxygen species and demonstrating DOX-induced ferroptosis occurrence [79]. LncRNA TINCR was demonstrated to function through recruiting IGF2BP1 to stabilize NLRP3 mRNA to trigger pyroptosis in DIC [29].

Additionally, HOXB-AS3 protects DOX-induced suppression in the proliferation of embryonic rat cardiomyocytes through targeting and downregulating miRNA-875-3p [80]. Some groups found that exosomal lncRNAs can be employed to alleviate DIC [82,84,85]. Exosome^MIF^ was reported as a promising anti-senescent strategy against DIC through transferring LncRNA–NEAT1, thus inhibiting miR-221-3p and leading to SIRT2 activation [82]. Chao Tian et al. reported that lncRNA MSTRG.58791.2 is possibly secreted by the bone marrow mesenchymal stem cells in exosomes (BMSC-Exos) and can alleviate DIC by suppressing inflammatory response and inflammation-related cell death [84]. Another study reported that Exosome^Hypoxia^ might serve as a potential therapeutic approach against DIC and that lncRNA-MALAT1/miR-92a-3p/ATG4a partially mediated the cardioprotective roles of exosome^Hypoxia^ in DOX-induced cardiac injury [85].

### 3.3. CircRNAs and DOX-Induced Cardiotoxicity

#### 3.3.1. Introduction to circRNAs

Circular RNAs (circRNAs) have a specific structure with exons and/or introns back-splicing formed loops, without 5′ end caps or 3′ end poly-A tails. They are mostly present in the cytoplasm and derived from exons [54]. Some circRNAs can be translated into proteins, but the majority are noncoding RNAs [131]. In addition to interacting with various proteins, circRNAs play regulatory roles in a manner as the competing endogenous RNAs (ceRNAs), thereby abolishing the effect of miRNAs on their target genes [92,94] (Figure 2). Although studies on circRNAs in the heart remain limited, previous studies have suggested that circRNAs may regulate cardiovascular development and disease. Given their extensive expression and sustained stability, circRNAs are highly appreciated as potential biomarkers of DIC [116,117] (Table 1).

#### 3.3.2. CircRNAs in Regulating DOX-Induced Cardiotoxicity

Circ-INSR is derived from the host gene encoding the insulin receptor (INSR). It is a conserved non-coding RNA. Lu et al. found that circ-INSR was significantly decreased in patients suffering from cardiotoxicity after DOX therapy, as well as in HL-1 mouse cardiomyocytes and hiPSC-CMs after DOX treatment [91]. Circ-INSR physically interacts with the single-stranded DNA-binding protein (SSBP1) to mediate its cardioprotective effect under DOX stress [91]. CircITCH is a tumor suppressor gene, whose host gene is ITCH (E3 ubiquitin protein ligase). CircITCH was downregulated in autopsy specimens from cancer patients with DOX-induced cardiomyopathy. It was found that CircITCH could act as an endogenous sponge to inhibit miR-330-5p, which consequently upregulated SIRT6, Survivin, and SERCA2a [92]. Wang et al. identified that circArhgap12 was upregulated upon DOX treatment and it regulated DIC by sponging miR-135a-5p [94]. Zeng et al. described a circular RNA, circ-Amotl1, which exhibited a protective role against DOX-induced cardiomyopathy by regulating protein Kinase B (Akt or PKB) phosphorylation [96]. Han et al. found that circular RNA (hsa_circ_0097435) was significantly overexpressed in patients with heart failure and DOX-treated human AC16 cells [97]. Li et al. found that circ-LTBP1 was increased in DOX-treated AC16 cells, which enhanced DOX-induced toxic effects through competitively sponging miR-107 and elevating adenylate cyclase 1 (ADCY1) [98]. Li et al. investigated the role of circ-SKA3 in DIC and found that circ-SKA3 was upregulated in DOX-treated AC16 cells and that knockdown of circ-SKA3 protected AC16 cells from DIC via the miR-1303/toll-like receptor 4 (TLR4) axis [99]. In addition, they revealed that circ-SKA3 could be packaged into exosomes and the exosomal circ-SKA3 level was elevated in DOX-treated AC16 cells, indicating that exosomal circ-SKA3 might be a potential biomarker and treatment target for preventing DIC [99].

CircNlgn can be translated into a small peptide Nlgn173. Increased expression of circNlgn led to compromised cardiac function and enhanced cardiac fibrosis by upregulating growth arrest and DNA damage inducible protein 45 b (Gadd45b), semaphorin-4C (Sema4C), and RAD50 and activating p38 and p-JNK via its translated Nlgn173 in transgenic hearts. Silencing circNlgn decreased the effects of DOX on cardiomyocytes and prevented DOX-induced expression of fibrotic genes [93]. Ji et al. reported that after DOX treatment, circPan3 was negatively regulated in cardiomyocytes by miR-31-5p via suppressing Quaking (QKI) [95]. Additionally, Xing et al. recently found dysregulation of three circRNAs, including circ_0015773, circ_0002106 and circ_0016006 in DIC [132]. Overall, further exploration is needed to identify circRNAs and characterize their functions and mechanisms in DIC.

## 4. Discussion

### 4.1. Non-Coding RNAs Bear Differential Regulatory Mechanisms in DIC

Currently, DOX-induced irreversible cardiac injury and heart failure remain a major obstacle for the clinical application of DOX in cancer therapy. Within the recent decade, accumulated evidence indicates that ncRNAs can regulate various aspects of DIC, including apoptosis, mitochondrial dysregulation, oxidative stress, DNA damage, autophagy, ferroptosis, pyroptosis, and ER stress. These ncRNA-mediated regulations occur at different gene expression levels including transcription, post-transcription, and translation.

MiRNAs, lncRNAs and circRNAs exert their regulatory function via differential mechanisms in DIC. MiRNAs primarily function at the post-transcriptional level, with mature miRNA binding to the 3′ untranslated region of target mRNA, which causes mRNA degradation and/or inhibition of translation. This miRNA-mRNA axis controls the protein expression level of specific miRNA target genes in DIC. Compared to miRNA, lncRNA can regulate DIC with more diverse mechanisms. For instance, lncRNAs can serve as scaffolds to recruit transcription factors or chromatin-modifying proteins to specific genes, thereby regulating their transcription [126]. LncRNAs can guide the recruitment of protein factors to associate with chromatin, thereby modifying chromatin structure to influence gene expression [126]. LncRNAs can also regulate translation by interacting with mRNAs or translation initiation factors [126]. Some lncRNAs may sequester mRNAs or affect the accessibility of mRNAs to the translation machinery, leading to translation repression or activation [126]. Since circRNAs are generated by back-splicing of host precursor mRNAs, circRNA expression levels are usually stable and abundant in cells. Although the direct involvement of circRNAs in transcriptional regulation remains poorly understood, some circRNAs can regulate the transcription of target genes through interacting with RNA-binding proteins. Also, circRNAs often act as miRNA sponge for inhibiting miRNA function, which sequesters miRNAs from interacting with target mRNAs. Therefore, circRNAs indirectly modulate translation by preventing miRNA-mRNA interaction. Notably, a small portion of lncRNAs and circRNAs could be translated into peptides [133,134] to regulate cellular processes. Furthermore, some ncRNAs, such as miR-21, may function differentially in different cardiomyopathies. MiR-21 exerts a cardioprotective role in DIC [70], whereas a profibrotic role in cardiac hypertrophy [135]. Moreover, the same ncRNA may display different functions at different stages or models of DIC. For instance, upregulated miR-146a was found in the acute DIC model and early stage of DIC [74], whereas it was downregulated in the chronic DIC model or late stage of DIC [66]. These indicate that ncRNAs might play temporal roles during the development of DIC. Taken together, non-coding RNAs form a complex regulatory network to control gene expression at multiple levels from transcriptional initiation to post-transcriptional regulation and translation. Further investigation into the molecular mechanisms of ncRNAs in DIC will be crucial for developing innovative therapeutic strategies to prevent or mitigate DIC, ultimately improving the outcomes of DOX in cancer therapy.

### 4.2. Non-Coding RNAs’ Impacts on DOX-Induced Antitumor Effect and Cardiotoxicity

Although small non-coding RNAs have been suggested as potential biomarkers and therapeutic candidates of DIC, it is notable that the interaction between tumor and heart might raise an additional layer of concern regarding non-coding RNA functions in affecting tumor growth and cardiomyocyte viability. Circulating small ncRNAs induced by DIC might affect tumor growth and metastasis, and tumor-secreted ncRNAs might influence the development of DIC, which could crosstalk to interfere with our understanding of ncRNAs’ roles in DIC, as well as for cancer therapeutics. Therefore, it is necessary to distinguish the protective ncRNAs in DIC, while not influencing the antitumor effect of DOX. In addition to DOX, other anticancer agents have also been reported to induce cardiac abnormalities and dysregulate the expression of ncRNAs, especially the recently emerged targeted cancer therapies and immune checkpoint inhibitors. However, research progress in this field is still limited, and further study is needed to identify the specific cardiac ncRNAs that are affected by those drugs. In addition, despite the significant progress on the mechanistic studies and prospective clinical applications of ncRNAs, the fundamental challenges of ncRNA-based therapeutics persist in targeted delivery systems and potential off-target consequences. Thus, achieving tissue-specific delivery with sustained on-target efficacy remains a primary issue.

### 4.3. Future Directions

Among the three types of ncRNAs, miRNAs are most extensively studied in DIC. Hence, future studies of lncRNAs and circRNAs might advance mechanistic study of DIC. Since most lncRNAs and circRNAs are tissue-specific and stably expressed, they could be employed as potential cardiac-specific biomarkers for diagnosis and therapeutic targets/approaches of DIC. For instance, our team recently reported that lncRNA *LIPTER* can mitigate metabolic syndrome-associated cardiomyopathy via preserving cardiac lipid metabolism [136]. As DOX could alter the metabolic homeostasis in the heart, preventing DOX-induced metabolic changes by ncRNAs could offer a new strategy to preserve cardiac function, and prevent heart failure in DIC. In summary, future studies should be focused on ncRNAs in the early diagnosis of DIC and the prevention of cardiac structural and functional impairment during DOX treatment, while preserving or even enhancing the antitumor efficacy of DOX, which will greatly expand the clinical applications of ncRNAs in DOX treatment. Understanding the dual roles of ncRNAs in both cardiac protection and tumor suppression will provide critical insights into optimizing DOX-mediated cancer therapy and mitigating its cardiotoxic effects.

## 5. Conclusions 

In conclusion, ncRNAs, including microRNAs, long non-coding RNAs, and circular RNAs, play critical role in regulating DIC. Due to their complex functions and interconnections in cancers and DIC, further mechanistic studies of ncRNAs in DIC will benefit the potential clinical applications of ncRNAs in mitigating and/or treating DIC.

## Figures and Tables

**Table 1:** NcRNAs in regulating DOX-induced cardiotoxicity.

NcRNA	Regulation	Targets	Biological Effects	Cell Type	Reference
**miRNAs**					
miR-15b-5p	Up	Bmpr1a	Mitochondrial dysfunction, ROS and apoptosis	Cardiomyocyte	[54]
miR-21-5p	Up	BTG2	Apoptosis	Cardiomyocyte	[55]
miR-22	Up	SIRT1	apoptosis	Cardiomyocyte	[56]
miR-23a-3p	Up	P53 and PGC-1α, Drp1	Mitochondrial dysfunction, ROS and apoptosis; fission	Cardiomyocyte	[57,58]
miR-29b	Down	Bax	Mitochondrial dysfunction and apoptosis	Cardiomyocyte	[59]
miR-30e	Down	β1AR, β2AR, Gia-2 and Bnip3L	ROS and apoptosis	Cardiomyocyte	[60]
miR-34a-5p	Up	SIRT1/P66shc	Apoptosis	Cardiomyocyte	[61,62]
miR-128	Up	SIRT1, PPARγ	Apoptosis	Cardiomyocyte	[63,64]
miR-130a	Up	PPARγ	Apoptosis	mESC-derived cardiac cells	[65]
miR-140-5p	Up	Nrf2 and SIRT2	Increase of ROS	Cardiomyocyte	[52]
miR-146a	Up/Down	ErbB4, TAF9b/P53	Mitochondrial dysfunction and apoptosis	Cardiomyocyte	[66,67]
miR-200a	Down	Nrf2	Ferroptosis	Cardiomyocyte	[68,69]
miR-208a-3p	Up	GATA4	Apoptosis	Cardiomyocyte	[70]
miR-212-3p/ miR-132-3p	Down	Fitm2	Apoptosis and atrophy	hiPSC-derived cardiomyocyte	[42]
miR-320a-3p	Up	VEGF-A	Reduced cardiac microvessel density and apoptosis	HUVEC	[71]
miR-378a-5p	Down	LDHA and PPIA	Energy metabolism disturbance and ER stress	Cardiomyocyte	[53]
miR-451-5p	Up	Cab39	ROS and apoptosis	Cardiomyocyte	[72]
miR-499-5p	Down	p21	Mitochondrial fission and apoptosis	Cardiomyocyte	[73,74]
miR-532-3p	Up	ARC	Mitochondrial fission and apoptosis	Cardiomyocyte	[48]
Let-7g-5p	Down	-	-	Cardiomyocyte	[75]
**LncRNAs**					
Lnc5745	Down	Rab2A	p53-related apoptosis	Cardiomyocyte	[76]
LncRNA NORAD	Down	Apaf-1, p53 and fission	fission, ROS and apoptosis	Cardiomyocyte	[77]
LINC00339	Up	miR-484	Apoptosis	Cardiomyocyte	[78]
LncRNA KCNQ1OT1	Modified	miR-7-5p	Ferroptosis	Cardiomyocyte	[79]
LncRNA HOXB-AS3	Down	miRNA-875-3p	Protecting cardiomyocytes	Cardiomyocyte	[80]
LncRNA CDML-1	Down	Drp1	Apoptosis	Cardiomyocyte	[81]
LncRNA NEAT1	-	miR-221-3p	Cardiac senescence	Cardiomyocyte	[82]
LncRNA SOX2-OT	Up	miR-942-5p/DP5	Apoptosis	Cardiomyocyte	[83]
ElncRNA MSTRG 58791.2	-	-	Inflammation	Cardiomyocyte	[84]
MALAT1	-	miR-92a-3p/ATG4a	Cardiac senescence	Cardiomyocyte	[85]
LncRNA RMRP	Down	PFN1/p53	Apoptosis	Cardiomyocyte	[86]
lincRNA-p21	Up	Wnt/β-catenin	Oxidative stress and cardiac senescence	Cardiomyocyte	[87]
LncRNA CHRF	Up	TGF-β/Smads and TGF-β/p38	Apoptosis	Cardiomyocyte	[88]
LncRNA FOXC2-AS1	Down	WISP1	cardiomyocyte viability	Cardiomyocyte	[89]
LncRNA Mhrt	Down	Nrf2	Apoptosis	Cardiomyocyte	[90]
LncRNA TINCR	Up	IGF2BP1/NLRP3	Pyroptosis	Cardiomyocyte	[29]
**CircRNAs**					
CircINSR	Down	SSBP1	Apoptosis	Cardiomyocyte	[91]
CircITCH	Down	miR-330-5p	Apoptosis	Cardiomyocyte	[92]
CircNlgn	Up	Nlgn173	Apoptosis and fibrosis	Cardiomyocyte	[93]
CircArhgap12	Up	miR-135-5p	Apoptosis	Cardiomyocyte	[94]
CircPan3	Down	-	Apoptosis	Cardiomyocyte	[95]
CircAmotl1	-	Akt	Apoptosis	Cardiomyocyte	[96]
Has_circ_0097435	Up	-	Apoptosis	Cardiomyocyte	[97]
Circ-LTBP1	Up	miR-107/ADCY1	Apoptosis and oxidative stress	Cardiomyocyte	[98]
Circ-SKA3	Up	miR-1303/TLR4	Apoptosis	Cardiomyocyte	[99]

## Data Availability

No new data were created or analyzed in this study.

## References

[1] Octavia Y., Tocchetti C.G., Gabrielson K.L., Janssens S., Crijns H.J., Moens A.L. (2012). Doxorubicin-induced cardiomyopathy: From molecular mechanisms to therapeutic strategies. J. Mol. Cell Cardiol..

[2] Reuter S.P., Soonpaa M.H., Field D., Simpson E., Lohe M.R.-v.d., Lee H.K., Sridhar A., Ware S.M., Green N., Li X. (2023). Cardiac Troponin I–Interacting Kinase Affects Cardiomyocyte S-Phase Activity but Not Cardiomyocyte Proliferation. Circulation.

[3] Rawat P.S., Jaiswal A., Khurana A., Bhatti J.S., Navik U. (2021). Doxorubicin-induced cardiotoxicity: An update on the molecular mechanism and novel therapeutic strategies for effective management. Biomed. Pharmacother..

[4] Zhang S., Liu X., Bawa-Khalfe T., Lu L.S., Lyu Y.L., Liu L.F., Yeh E.T. (2012). Identification of the molecular basis of doxorubicin-induced cardiotoxicity. Nat. Med..

[5] Davies K.J., Doroshow J.H. (1986). Redox cycling of anthracyclines by cardiac mitochondria. I. Anthracycline radical formation by NADH dehydrogenase. J. Biol. Chem..

[6] Green P.S., Leeuwenburgh C. (2002). Mitochondrial dysfunction is an early indicator of doxorubicin-induced apoptosis. Biochim. Biophys. Acta.

[7] Arai M., Tomaru K., Takizawa T., Sekiguchi K., Yokoyama T., Suzuki T., Nagai R. (1998). Sarcoplasmic reticulum genes are selectively down-regulated in cardiomyopathy produced by doxorubicin in rabbits. J. Mol. Cell Cardiol..

[8] Arai M., Yoguchi A., Takizawa T., Yokoyama T., Kanda T., Kurabayashi M., Nagai R. (2000). Mechanism of doxorubicin-induced inhibition of sarcoplasmic reticulum Ca(2+)-ATPase gene transcription. Circ. Res..

[9] Li Y., Yan J., Yang P. (2024). The mechanism and therapeutic strategies in doxorubicin-induced cardiotoxicity: Role of programmed cell death. Cell Stress. Chaperones.

[10] Fa H.G., Chang W.G., Zhang X.J., Xiao D.D., Wang J.X. (2021). Noncoding RNAs in doxorubicin-induced cardiotoxicity and their potential as biomarkers and therapeutic targets. Acta Pharmacol. Sin..

[11] Vedam K., Nishijima Y., Druhan L.J., Khan M., Moldovan N.I., Zweier J.L., Ilangovan G. (2010). Role of heat shock factor-1 activation in the doxorubicin-induced heart failure in mice. Am. J. Physiol. Heart Circ. Physiol..

[12] Chen Y., Huang T., Shi W., Fang J., Deng H., Cui G. (2020). Potential targets for intervention against doxorubicin-induced cardiotoxicity based on genetic studies: A systematic review of the literature. J. Mol. Cell Cardiol..

[13] Ghigo A., Li M., Hirsch E. (2016). New signal transduction paradigms in anthracycline-induced cardiotoxicity. Biochim. Biophys. Acta.

[14] Parker M.A., King V., Howard K.P. (2001). Nuclear magnetic resonance study of doxorubicin binding to cardiolipin containing magnetically oriented phospholipid bilayers. Biochim. Biophys. Acta.

[15] Aryal B., Rao V.A. (2016). Deficiency in Cardiolipin Reduces Doxorubicin-Induced Oxidative Stress and Mitochondrial Damage in Human B-Lymphocytes. PLoS ONE.

[16] Schlame M., Rua D., Greenberg M.L. (2000). The biosynthesis and functional role of cardiolipin. Prog. Lipid Res..

[17] Wu B.B., Leung K.T., Poon E.N. (2022). Mitochondrial-Targeted Therapy for Doxorubicin-Induced Cardiotoxicity. Int. J. Mol. Sci..

[18] Carvalho R.A., Sousa R.P., Cadete V.J., Lopaschuk G.D., Palmeira C.M., Bjork J.A., Wallace K.B. (2010). Metabolic remodeling associated with subchronic doxorubicin cardiomyopathy. Toxicology.

[19] Aries A., Paradis P., Lefebvre C., Schwartz R.J., Nemer M. (2004). Essential role of GATA-4 in cell survival and drug-induced cardiotoxicity. Proc. Natl. Acad. Sci. USA.

[20] Suzuki Y.J. (2003). Stress-induced activation of GATA-4 in cardiac muscle cells. Free Radic. Biol. Med..

[21] Wu L., Zhang Y., Wang G., Ren J. (2024). Molecular Mechanisms and Therapeutic Targeting of Ferroptosis in Doxorubicin-Induced Cardiotoxicity. JACC Basic. Transl. Sci..

[22] Dixon S.J., Lemberg K.M., Lamprecht M.R., Skouta R., Zaitsev E.M., Gleason C.E., Patel D.N., Bauer A.J., Cantley A.M., Yang W.S. (2012). Ferroptosis: An iron-dependent form of nonapoptotic cell death. Cell.

[23] Yan H.F., Zou T., Tuo Q.Z., Xu S., Li H., Belaidi A.A., Lei P. (2021). Ferroptosis: Mechanisms and links with diseases. Signal Transduct. Target. Ther..

[24] Yi X., Wang Q., Zhang M., Shu Q., Zhu J. (2024). Ferroptosis: A novel therapeutic target of natural products against doxorubicin-induced cardiotoxicity. Biomed. Pharmacother..

[25] Conrad M., Proneth B. (2019). Broken hearts: Iron overload, ferroptosis and cardiomyopathy. Cell Res..

[26] Fang X., Wang H., Han D., Xie E., Yang X., Wei J., Gu S., Gao F., Zhu N., Yin X. (2019). Ferroptosis as a target for protection against cardiomyopathy. Proc. Natl. Acad. Sci. USA.

[27] Wang Y., Yan S., Liu X., Deng F., Wang P., Yang L., Hu L., Huang K., He J. (2022). PRMT4 promotes ferroptosis to aggravate doxorubicin-induced cardiomyopathy via inhibition of the Nrf2/GPX4 pathway. Cell Death Differ..

[28] Cookson B.T., Brennan M.A. (2001). Pro-inflammatory programmed cell death. Trends Microbiol..

[29] Meng L., Lin H., Zhang J., Lin N., Sun Z., Gao F., Luo H., Ni T., Luo W., Chi J. (2019). Doxorubicin induces cardiomyocyte pyroptosis via the TINCR-mediated posttranscriptional stabilization of NLR family pyrin domain containing 3. J. Mol. Cell Cardiol..

[30] Christidi E., Brunham L.R. (2021). Regulated cell death pathways in doxorubicin-induced cardiotoxicity. Cell Death Dis..

[31] Ye B., Shi X., Xu J., Dai S., Xu J., Fan X., Han B., Han J. (2022). Gasdermin D mediates doxorubicin-induced cardiomyocyte pyroptosis and cardiotoxicity via directly binding to doxorubicin and changes in mitochondrial damage. Transl. Res..

[32] Dai S., Chen Y., Fan X., Han J., Zhong L., Zhang Y., Liu Q., Lin J., Huang W., Su L. (2023). Emodin attenuates cardiomyocyte pyroptosis in doxorubicin-induced cardiotoxicity by directly binding to GSDMD. Phytomedicine.

[33] Li Z., Song Y., Liu L., Hou N., An X., Zhan D., Li Y., Zhou L., Li P., Yu L. (2017). miR-199a impairs autophagy and induces cardiac hypertrophy through mTOR activation. Cell Death Differ..

[34] Lekli I., Haines D.D., Balla G., Tosaki A. (2017). Autophagy: An adaptive physiological countermeasure to cellular senescence and ischaemia/reperfusion-associated cardiac arrhythmias. J. Cell Mol. Med..

[35] Kobayashi S., Volden P., Timm D., Mao K., Xu X., Liang Q. (2010). Transcription factor GATA4 inhibits doxorubicin-induced autophagy and cardiomyocyte death. J. Biol. Chem..

[36] Velez J.M., Miriyala S., Nithipongvanitch R., Noel T., Plabplueng C.D., Oberley T., Jungsuwadee P., Van Remmen H., Vore M., St Clair D.K. (2011). p53 Regulates oxidative stress-mediated retrograde signaling: A novel mechanism for chemotherapy-induced cardiac injury. PLoS ONE.

[37] Tran S., Fairlie W.D., Lee E.F. (2021). BECLIN1: Protein Structure, Function and Regulation. Cells.

[38] He H., Luo Y., Qiao Y., Zhang Z., Yin D., Yao J., You J., He M. (2018). Curcumin attenuates doxorubicin-induced cardiotoxicity via suppressing oxidative stress and preventing mitochondrial dysfunction mediated by 14-3-3gamma. Food Funct..

[39] Bartlett J.J., Trivedi P.C., Yeung P., Kienesberger P.C., Pulinilkunnil T. (2016). Doxorubicin impairs cardiomyocyte viability by suppressing transcription factor EB expression and disrupting autophagy. Biochem. J..

[40] Takla M., Keshri S., Rubinsztein D.C. (2023). The post-translational regulation of transcription factor EB (TFEB) in health and disease. EMBO Rep..

[41] Zhu W., Soonpaa M.H., Chen H., Shen W., Payne R.M., Liechty E.A., Caldwell R.L., Shou W., Field L.J. (2009). Acute doxorubicin cardiotoxicity is associated with p53-induced inhibition of the mammalian target of rapamycin pathway. Circulation.

[42] Chen M.B., Wu X.Y., Gu J.H., Guo Q.T., Shen W.X., Lu P.H. (2011). Activation of AMP-activated protein kinase contributes to doxorubicin-induced cell death and apoptosis in cultured myocardial H9c2 cells. Cell Biochem. Biophys..

[43] Koleini N., Kardami E. (2017). Autophagy and mitophagy in the context of doxorubicin-induced cardiotoxicity. Oncotarget.

[44] Renu K., Abilash V.G., Tirupathi Pichiah P.B., Arunachalam S. (2018). Molecular mechanism of doxorubicin-induced cardiomyopathy—An update. Eur. J. Pharmacol..

[45] Ambros V. (2001). microRNAs: Tiny regulators with great potential. Cell.

[46] Hutvagner G., Zamore P.D. (2002). A microRNA in a multiple-turnover RNAi enzyme complex. Science.

[47] Bartel D.P. (2004). MicroRNAs: Genomics, biogenesis, mechanism, and function. Cell.

[48] Wang J.X., Zhang X.J., Feng C., Sun T., Wang K., Wang Y., Zhou L.Y., Li P.F. (2015). MicroRNA-532-3p regulates mitochondrial fission through targeting apoptosis repressor with caspase recruitment domain in doxorubicin cardiotoxicity. Cell Death Dis..

[49] Zhao Y., Ransom J.F., Li A., Vedantham V., von Drehle M., Muth A.N., Tsuchihashi T., McManus M.T., Schwartz R.J., Srivastava D. (2007). Dysregulation of cardiogenesis, cardiac conduction, and cell cycle in mice lacking miRNA-1-2. Cell.

[50] van Rooij E., Sutherland L.B., Thatcher J.E., DiMaio J.M., Naseem R.H., Marshall W.S., Hill J.A., Olson E.N. (2008). Dysregulation of microRNAs after myocardial infarction reveals a role of miR-29 in cardiac fibrosis. Proc. Natl. Acad. Sci. USA.

[51] Elia L., Contu R., Quintavalle M., Varrone F., Chimenti C., Russo M.A., Cimino V., De Marinis L., Frustaci A., Catalucci D. (2009). Reciprocal regulation of microRNA-1 and insulin-like growth factor-1 signal transduction cascade in cardiac and skeletal muscle in physiological and pathological conditions. Circulation.

[52] Zhao L., Qi Y., Xu L., Tao X., Han X., Yin L., Peng J. (2018). MicroRNA-140-5p aggravates doxorubicin-induced cardiotoxicity by promoting myocardial oxidative stress via targeting Nrf2 and Sirt2. Redox Biol..

[53] Wang Y., Zhang Q., Wei C., Zhao L., Guo X., Cui X., Shao L., Long J., Gu J., Zhao M. (2018). MiR-378 modulates energy imbalance and apoptosis of mitochondria induced by doxorubicin. Am. J. Transl. Res..

[54] Wan G.X., Cheng L., Qin H.L., Zhang Y.Z., Wang L.Y., Zhang Y.G. (2019). MiR-15b-5p is Involved in Doxorubicin-Induced Cardiotoxicity via Inhibiting Bmpr1a Signal in H9c2 Cardiomyocyte. Cardiovasc. Toxicol..

[55] Tong Z., Jiang B., Wu Y., Liu Y., Li Y., Gao M., Jiang Y., Lv Q., Xiao X. (2015). MiR-21 Protected Cardiomyocytes against Doxorubicin-Induced Apoptosis by Targeting BTG2. Int. J. Mol. Sci..

[56] Xu C., Liu C.H., Zhang D.L. (2020). MicroRNA-22 inhibition prevents doxorubicin-induced cardiotoxicity via upregulating SIRT1. Biochem. Biophys. Res. Commun..

[57] Li J., Aung L.H., Long B., Qin D., An S., Li P. (2015). miR-23a binds to p53 and enhances its association with miR-128 promoter. Sci. Rep..

[58] Du J., Hang P., Pan Y., Feng B., Zheng Y., Chen T., Zhao L., Du Z. (2019). Inhibition of miR-23a attenuates doxorubicin-induced mitochondria-dependent cardiomyocyte apoptosis by targeting the PGC-1alpha/Drp1 pathway. Toxicol. Appl. Pharmacol..

[59] Jing X., Yang J., Jiang L., Chen J., Wang H. (2018). MicroRNA-29b Regulates the Mitochondria-Dependent Apoptotic Pathway by Targeting Bax in Doxorubicin Cardiotoxicity. Cell Physiol. Biochem..

[60] Roca-Alonso L., Castellano L., Mills A., Dabrowska A.F., Sikkel M.B., Pellegrino L., Jacob J., Frampton A.E., Krell J., Coombes R.C. (2015). Myocardial MiR-30 downregulation triggered by doxorubicin drives alterations in beta-adrenergic signaling and enhances apoptosis. Cell Death Dis..

[61] Piegari E., Russo R., Cappetta D., Esposito G., Urbanek K., Dell’Aversana C., Altucci L., Berrino L., Rossi F., De Angelis A. (2016). MicroRNA-34a regulates doxorubicin-induced cardiotoxicity in rat. Oncotarget.

[62] Zhu J.N., Fu Y.H., Hu Z.Q., Li W.Y., Tang C.M., Fei H.W., Yang H., Lin Q.X., Gou D.M., Wu S.L. (2017). Activation of miR-34a-5p/Sirt1/p66shc pathway contributes to doxorubicin-induced cardiotoxicity. Sci. Rep..

[63] Adlakha Y.K., Saini N. (2013). miR-128 exerts pro-apoptotic effect in a p53 transcription-dependent and -independent manner via PUMA-Bak axis. Cell Death Dis..

[64] Zhang W.B., Zheng Y.F., Wu Y.G. (2021). Inhibition of miR-128-3p Attenuated Doxorubicin-Triggered Acute Cardiac Injury in Mice by the Regulation of PPAR-gamma. PPAR Res..

[65] Pakravan G., Foroughmand A.M., Peymani M., Ghaedi K., Hashemi M.S., Hajjari M., Nasr-Esfahani M.H. (2018). Downregulation of miR-130a, antagonized doxorubicin-induced cardiotoxicity via increasing the PPARgamma expression in mESCs-derived cardiac cells. Cell Death Dis..

[66] Horie T., Ono K., Nishi H., Nagao K., Kinoshita M., Watanabe S., Kuwabara Y., Nakashima Y., Takanabe-Mori R., Nishi E. (2010). Acute doxorubicin cardiotoxicity is associated with miR-146a-induced inhibition of the neuregulin-ErbB pathway. Cardiovasc. Res..

[67] Pan J.A., Tang Y., Yu J.Y., Zhang H., Zhang J.F., Wang C.Q., Gu J. (2019). miR-146a attenuates apoptosis and modulates autophagy by targeting TAF9b/P53 pathway in doxorubicin-induced cardiotoxicity. Cell Death Dis..

[68] Lin Z., Wang J. (2023). Taxifolin protects against doxorubicin-induced cardiotoxicity and ferroptosis by adjusting microRNA-200a-mediated Nrf2 signaling pathway. Heliyon.

[69] Hu X., Liu H., Wang Z., Hu Z., Li L. (2019). miR-200a Attenuated Doxorubicin-Induced Cardiotoxicity through Upregulation of Nrf2 in Mice. Oxid. Med. Cell Longev..

[70] Tony H., Yu K., Qiutang Z. (2015). MicroRNA-208a Silencing Attenuates Doxorubicin Induced Myocyte Apoptosis and Cardiac Dysfunction. Oxid. Med. Cell Longev..

[71] Yin Z., Zhao Y., Li H., Yan M., Zhou L., Chen C., Wang D.W. (2016). miR-320a mediates doxorubicin-induced cardiotoxicity by targeting VEGF signal pathway. Aging.

[72] Li J., Wan W., Chen T., Tong S., Jiang X., Liu W. (2019). miR-451 Silencing Inhibited Doxorubicin Exposure-Induced Cardiotoxicity in Mice. Biomed. Res. Int..

[73] Wang J.X., Jiao J.Q., Li Q., Long B., Wang K., Liu J.P., Li Y.R., Li P.F. (2011). miR-499 regulates mitochondrial dynamics by targeting calcineurin and dynamin-related protein-1. Nat. Med..

[74] Wan Q., Xu T., Ding W., Zhang X., Ji X., Yu T., Yu W., Lin Z., Wang J. (2018). miR-499-5p Attenuates Mitochondrial Fission and Cell Apoptosis via p21 in Doxorubicin Cardiotoxicity. Front. Genet..

[75] Fu J., Peng C., Wang W., Jin H., Tang Q., Wei X. (2012). Let-7 g is involved in doxorubicin induced myocardial injury. Env. Toxicol. Pharmacol..

[76] Cai H., Tian P., Ju J., Wang T., Chen X., Wang K., Wang F., Yu X., Wang S., Wang Y. (2022). Long noncoding RNA NONMMUT015745 inhibits doxorubicin-mediated cardiomyocyte apoptosis by regulating Rab2A-p53 axis. Cell Death Discov..

[77] Guan X., Wang Y., Li W., Liu X., Jiang J., Bian W., Xu C., Sun Y., Zhang C. (2023). The effects and mechanism of LncRNA NORAD on doxorubicin-induced cardiotoxicity. Toxicology.

[78] Li J., Li L., Li X., Wu S. (2018). Long noncoding RNA LINC00339 aggravates doxorubicin-induced cardiomyocyte apoptosis by targeting MiR-484. Biochem. Biophys. Res. Commun..

[79] Zhuang S., Ma Y., Zeng Y., Lu C., Yang F., Jiang N., Ge J., Ju H., Zhong C., Wang J. (2023). METTL14 promotes doxorubicin-induced cardiomyocyte ferroptosis by regulating the KCNQ1OT1-miR-7-5p-TFRC axis. Cell Biol. Toxicol..

[80] Lu Q., Huo J., Liu P., Bai L., Ma A. (2020). lncRNA HOXB-AS3 protects doxorubicin-induced cardiotoxicity by targeting miRNA-875-3p. Exp. Ther. Med..

[81] Aung L.H.H., Chen X., Cueva Jumbo J.C., Li Z., Wang S.Y., Zhao C., Liu Z., Wang Y., Li P. (2021). Cardiomyocyte mitochondrial dynamic-related lncRNA 1 (CMDL-1) may serve as a potential therapeutic target in doxorubicin cardiotoxicity. Mol. Ther. Nucleic Acids.

[82] Zhuang L., Xia W., Chen D., Ye Y., Hu T., Li S., Hou M. (2020). Exosomal LncRNA-NEAT1 derived from MIF-treated mesenchymal stem cells protected against doxorubicin-induced cardiac senescence through sponging miR-221-3p. J. Nanobiotechnology.

[83] Wang H., Lin X., Li J., Zeng G., Xu T. (2021). Long Noncoding RNA SOX2-OT Aggravates Doxorubicin-Induced Apoptosis of Cardiomyocyte by Targeting miR-942-5p/DP5. Drug Des. Devel Ther..

[84] Tian C., Yang Y., Li B., Liu M., He X., Zhao L., Song X., Yu T., Chu X.M. (2022). Doxorubicin-Induced Cardiotoxicity May Be Alleviated by Bone Marrow Mesenchymal Stem Cell-Derived Exosomal lncRNA via Inhibiting Inflammation. J. Inflamm. Res..

[85] Xia W., Chen H., Xie C., Hou M. (2020). Long-noncoding RNA MALAT1 sponges microRNA-92a-3p to inhibit doxorubicin-induced cardiac senescence by targeting ATG4a. Aging.

[86] Li J., Zhou L., Jiang Y., Gao H., Maierhaba T., Gong H. (2023). Long noncoding RNA RMRP ameliorates doxorubicin-induced apoptosis by interacting with PFN1 in a P53-Dependent manner. Mol. Cell Probes.

[87] Xie Z., Xia W., Hou M. (2018). Long intergenic non-coding RNA-p21 mediates cardiac senescence via the Wnt/beta-catenin signaling pathway in doxorubicin-induced cardiotoxicity. Mol. Med. Rep..

[88] Chen L., Yan K.P., Liu X.C., Wang W., Li C., Li M., Qiu C.G. (2018). Valsartan regulates TGF-beta/Smads and TGF-beta/p38 pathways through lncRNA CHRF to improve doxorubicin-induced heart failure. Arch. Pharm. Res..

[89] Zhang S., Yuan Y., Zhang Z., Guo J., Li J., Zhao K., Qin Y., Qiu C. (2019). LncRNA FOXC2-AS1 protects cardiomyocytes from doxorubicin-induced cardiotoxicity through activation of WNT1-inducible signaling pathway protein-1. Biosci. Biotechnol. Biochem..

[90] Li H.Q., Wu Y.B., Yin C.S., Chen L., Zhang Q., Hu L.Q. (2016). Obestatin attenuated doxorubicin-induced cardiomyopathy via enhancing long noncoding Mhrt RNA expression. Biomed. Pharmacother..

[91] Lu D., Chatterjee S., Xiao K., Riedel I., Huang C.K., Costa A., Cushman S., Neufeldt D., Rode L., Schmidt A. (2022). A circular RNA derived from the insulin receptor locus protects against doxorubicin-induced cardiotoxicity. Eur. Heart J..

[92] Han D., Wang Y., Wang Y., Dai X., Zhou T., Chen J., Tao B., Zhang J., Cao F. (2020). The Tumor-Suppressive Human Circular RNA CircITCH Sponges miR-330-5p to Ameliorate Doxorubicin-Induced Cardiotoxicity Through Upregulating SIRT6, Survivin, and SERCA2a. Circ. Res..

[93] Xu J., Du W.W., Wu N., Li F., Li X., Xie Y., Wang S., Yang B.B. (2022). The circular RNA circNlgnmediates doxorubicin-inducedcardiac remodeling and fibrosis. Mol. Ther. Nucleic Acids.

[94] Wang X., Cheng Z., Xu J., Feng M., Zhang H., Zhang L., Qian L. (2021). Circular RNA Arhgap12 modulates doxorubicin-induced cardiotoxicity by sponging miR-135a-5p. Life Sci..

[95] Ji X., Ding W., Xu T., Zheng X., Zhang J., Liu M., Liu G., Wang J. (2020). MicroRNA-31-5p attenuates doxorubicin-induced cardiotoxicity via quaking and circular RNA Pan3. J. Mol. Cell Cardiol..

[96] Zeng Y., Du W.W., Wu Y., Yang Z., Awan F.M., Li X., Yang W., Zhang C., Yang Q., Yee A. (2017). A Circular RNA Binds To and Activates AKT Phosphorylation and Nuclear Localization Reducing Apoptosis and Enhancing Cardiac Repair. Theranostics.

[97] Han J., Zhang L., Hu L., Yu H., Xu F., Yang B., Zhang R., Zhang Y., An Y. (2020). Circular RNA-Expression Profiling Reveals a Potential Role of Hsa_circ_0097435 in Heart Failure via Sponging Multiple MicroRNAs. Front. Genet..

[98] Li C., Zhang L., Bu X., Wang J., Li L., Yang Z. (2022). Circ-LTBP1 is involved in doxorubicin-induced intracellular toxicity in cardiomyocytes via miR-107/ADCY1 signal. Mol. Cell Biochem..

[99] Li B., Cai X., Wang Y., Zhu H., Zhang P., Jiang P., Yang X., Sun J., Hong L., Shao L. (2021). Circ-SKA3 Enhances Doxorubicin Toxicity in AC16 Cells Through miR-1303/TLR4 Axis. Int. Heart J..

[100] Arola O.J., Saraste A., Pulkki K., Kallajoki M., Parvinen M., Voipio-Pulkki L.M. (2000). Acute doxorubicin cardiotoxicity involves cardiomyocyte apoptosis. Cancer Res..

[101] Delpy E., Hatem S.N., Andrieu N., de Vaumas C., Henaff M., Rucker-Martin C., Jaffrezou J.P., Laurent G., Levade T., Mercadier J.J. (1999). Doxorubicin induces slow ceramide accumulation and late apoptosis in cultured adult rat ventricular myocytes. Cardiovasc. Res..

[102] Kawano I., Adamcova M. (2022). MicroRNAs in doxorubicin-induced cardiotoxicity: The DNA damage response. Front. Pharmacol..

[103] De Angelis A., Piegari E., Cappetta D., Russo R., Esposito G., Ciuffreda L.P., Ferraiolo F.A., Frati C., Fagnoni F., Berrino L. (2015). SIRT1 activation rescues doxorubicin-induced loss of functional competence of human cardiac progenitor cells. Int. J. Cardiol..

[104] Ren Y., Sun C., Sun Y., Tan H., Wu Y., Cui B., Wu Z. (2009). PPAR gamma protects cardiomyocytes against oxidative stress and apoptosis via Bcl-2 upregulation. Vasc. Pharmacol..

[105] Goormaghtigh E., Chatelain P., Caspers J., Ruysschaert J.M. (1980). Evidence of a complex between adriamycin derivatives and cardiolipin: Possible role in cardiotoxicity. Biochem. Pharmacol..

[106] Ashley N., Poulton J. (2009). Mitochondrial DNA is a direct target of anti-cancer anthracycline drugs. Biochem. Biophys. Res. Commun..

[107] Childs A.C., Phaneuf S.L., Dirks A.J., Phillips T., Leeuwenburgh C. (2002). Doxorubicin treatment in vivo causes cytochrome C release and cardiomyocyte apoptosis, as well as increased mitochondrial efficiency, superoxide dismutase activity, and Bcl-2:Bax ratio. Cancer Res..

[108] Sprenger H.G., Langer T. (2019). The Good and the Bad of Mitochondrial Breakups. Trends Cell Biol..

[109] Quiles J.M., Gustafsson A.B. (2022). The role of mitochondrial fission in cardiovascular health and disease. Nat. Rev. Cardiol..

[110] Liang X., Wang S., Wang L., Ceylan A.F., Ren J., Zhang Y. (2020). Mitophagy inhibitor liensinine suppresses doxorubicin-induced cardiotoxicity through inhibition of Drp1-mediated maladaptive mitochondrial fission. Pharmacol. Res..

[111] Doughan A.K., Harrison D.G., Dikalov S.I. (2008). Molecular mechanisms of angiotensin II-mediated mitochondrial dysfunction: Linking mitochondrial oxidative damage and vascular endothelial dysfunction. Circ. Res..

[112] Mukhopadhyay P., Rajesh M., Batkai S., Kashiwaya Y., Hasko G., Liaudet L., Szabo C., Pacher P. (2009). Role of superoxide, nitric oxide, and peroxynitrite in doxorubicin-induced cell death in vivo and in vitro. Am. J. Physiol. Heart Circ. Physiol..

[113] Lebrecht D., Setzer B., Ketelsen U.P., Haberstroh J., Walker U.A. (2003). Time-dependent and tissue-specific accumulation of mtDNA and respiratory chain defects in chronic doxorubicin cardiomyopathy. Circulation.

[114] Wallace K.B., Sardao V.A., Oliveira P.J. (2020). Mitochondrial Determinants of Doxorubicin-Induced Cardiomyopathy. Circ. Res..

[115] Regula K.M., Ens K., Kirshenbaum L.A. (2002). Inducible expression of BNIP3 provokes mitochondrial defects and hypoxia-mediated cell death of ventricular myocytes. Circ. Res..

[116] Siveski-Iliskovic N., Kaul N., Singal P.K. (1994). Probucol promotes endogenous antioxidants and provides protection against adriamycin-induced cardiomyopathy in rats. Circulation.

[117] Yen H.C., Oberley T.D., Vichitbandha S., Ho Y.S., St Clair D.K. (1996). The protective role of manganese superoxide dismutase against adriamycin-induced acute cardiac toxicity in transgenic mice. J. Clin. Investig..

[118] Deniaud A., Sharaf el dein O., Maillier E., Poncet D., Kroemer G., Lemaire C., Brenner C. (2008). Endoplasmic reticulum stress induces calcium-dependent permeability transition, mitochondrial outer membrane permeabilization and apoptosis. Oncogene.

[119] Dodd D.A., Atkinson J.B., Olson R.D., Buck S., Cusack B.J., Fleischer S., Boucek R.J. (1993). Doxorubicin cardiomyopathy is associated with a decrease in calcium release channel of the sarcoplasmic reticulum in a chronic rabbit model. J. Clin. Investig..

[120] Gupta S.K., Garg A., Avramopoulos P., Engelhardt S., Streckfuss-Bomeke K., Batkai S., Thum T. (2019). miR-212/132 Cluster Modulation Prevents Doxorubicin-Mediated Atrophy and Cardiotoxicity. Mol. Ther..

[121] Luu A.Z., Chowdhury B., Al-Omran M., Teoh H., Hess D.A., Verma S. (2018). Role of Endothelium in Doxorubicin-Induced Cardiomyopathy. JACC Basic. Transl. Sci..

[122] Octavia Y., Kararigas G., de Boer M., Chrifi I., Kietadisorn R., Swinnen M., Duimel H., Verheyen F.K., Brandt M.M., Fliegner D. (2017). Folic acid reduces doxorubicin-induced cardiomyopathy by modulating endothelial nitric oxide synthase. J. Cell Mol. Med..

[123] Fernandez-Fernandez A., Carvajal D.A., Lei T., McGoron A.J. (2014). Chemotherapy-induced changes in cardiac capillary permeability measured by fluorescent multiple indicator dilution. Ann. Biomed. Eng..

[124] Wilkinson E.L., Sidaway J.E., Cross M.J. (2016). Cardiotoxic drugs Herceptin and doxorubicin inhibit cardiac microvascular endothelial cell barrier formation resulting in increased drug permeability. Biol. Open.

[125] Mattick J.S., Amaral P.P., Carninci P., Carpenter S., Chang H.Y., Chen L.L., Chen R., Dean C., Dinger M.E., Fitzgerald K.A. (2023). Long non-coding RNAs: Definitions, functions, challenges and recommendations. Nat. Rev. Mol. Cell Biol..

[126] Zhang X., Wang W., Zhu W., Dong J., Cheng Y., Yin Z., Shen F. (2019). Mechanisms and Functions of Long Non-Coding RNAs at Multiple Regulatory Levels. Int. J. Mol. Sci..

[127] Ma L., Zhang H., Zhang Y., Li H., An M., Zhao B., Ding H., Xu J., Shang H., Han X. (2021). Integrated analysis of lncRNA, miRNA and mRNA profiles reveals potential lncRNA functions during early HIV infection. J. Transl. Med..

[128] Wu X.S., Wang F., Li H.F., Hu Y.P., Jiang L., Zhang F., Li M.L., Wang X.A., Jin Y.P., Zhang Y.J. (2024). Author Correction: LncRNA-PAGBC acts as a microRNA sponge and promotes gallbladder tumorigenesis. EMBO Rep..

[129] Anderson K.M., Anderson D.M. (2022). LncRNAs at the heart of development and disease. Mamm. Genome.

[130] Thum T., Condorelli G. (2015). Long noncoding RNAs and microRNAs in cardiovascular pathophysiology. Circ. Res..

[131] Schneider T., Bindereif A. (2017). Circular RNAs: Coding or noncoding?. Cell Res..

[132] Xing X., Tan Z., Zhi X., Sun H., Yang J., Li L., Liu Y., Wang L., Dong Z., Guo H. (2022). Integrating analysis of circular RNA and mRNA expression profiles in doxorubicin induced cardiotoxicity mice. J. Appl. Toxicol..

[133] Pang Y., Liu Z., Han H., Wang B., Li W., Mao C., Liu S. (2020). Peptide SMIM30 promotes HCC development by inducing SRC/YES1 membrane anchoring and MAPK pathway activation. J. Hepatol..

[134] Lu P., Fan J., Li B., Wang X., Song M. (2024). A novel protein encoded by circLARP1B promotes the proliferation and migration of vascular smooth muscle cells by suppressing cAMP signaling. Atherosclerosis.

[135] Surina S., Fontanella R.A., Scisciola L., Marfella R., Paolisso G., Barbieri M. (2021). miR-21 in Human Cardiomyopathies. Front. Cardiovasc. Med..

[136] Han L., Huang D., Wu S., Liu S., Wang C., Sheng Y., Lu X., Broxmeyer H.E., Wan J., Yang L. (2023). Lipid droplet-associated lncRNA LIPTER preserves cardiac lipid metabolism. Nat. Cell Biol..

